# A retrospective review of calls to the Poisons Information Helpline of the Western Cape during the first 6 months of the COVID-19 pandemic in South Africa

**DOI:** 10.4102/sajid.v37i1.391

**Published:** 2022-03-30

**Authors:** Catharina E. du Plessis, Farah Mohamed, Cindy R. Stephen, Helmuth Reuter, Gonwayne Voigt, Daniel J. van Hoving, Carine J. Marks

**Affiliations:** 1 Division of Clinical Pharmacology, Faculty of Medicine and Health Sciences, Stellenbosch University, Cape Town, South Africa; 2 Department of Paediatrics and Child Health, University of Cape Town, Cape Town, South Africa; 3 Department of Medicine, Faculty of Medicine and Health Science, Stellenbosch University, Cape Town, South Africa; 4 Division of Emergency Medicine, Faculty of Medicine and Health Science, Stellenbosch University, Cape Town, South Africa

**Keywords:** COVID-19, poison helpline, poison exposures, pharmaceuticals, antiseptics and disinfectants

## Abstract

**Background:**

Since the start of the coronavirus disease 2019 (COVID-19) pandemic, poison centres worldwide have reported an increase in exposures to chemicals used for infection prevention. Increased availability and use could lead to an increase in exposures. Potential effects on a South African Poison Information Helpline were unknown, therefore a study was performed to describe changes in call volume and profile of poison exposures.

**Methods:**

A retrospective analysis was conducted on an observational database of telephone enquiries. All human-related poisoning exposure call data collected from 01 March to 31 August during 2018, 2019 and 2020 were extracted and analysed. Summary statistics were used to describe all variables.

**Results:**

The total number of calls were 5137, 5508, and 5181 in 2018, 2019, 2020, respectively. The monthly call number during 2020 was mostly less than in 2019. More calls were received from the public calls (39.4% vs 33.1%) and for accidental exposures (65.6% vs 62.3%) increased during 2020 compared to 2019. Exposures to pharmaceuticals decreased by 14.8% from 2019 to 2020, while exposures to eucalyptus oil more than doubled from 21 in 2019 to 43 during 2020. Exposures to antiseptics and disinfectants increased by 60.4%, mainly due to hand sanitisers exposure which showed a 26-fold increase from 2019 (*n* = 6) to 2020 (*n* = 156).

**Conclusion:**

A change in the profile of poison exposures was observed during the COVID-19 pandemic. Lockdown regulations and greater availability of antiseptics and disinfectants probably led to the increase in exposures. Although symptoms were mostly mild, the public should be educated on safe storage and proper use of all chemicals.

## Introduction

The World Health Organization (WHO) declared the recent outbreak of the novel coronavirus disease 2019 (COVID-19) a worldwide pandemic in March 2020.^1^ Subsequently in South Africa, a National State of Disaster was declared on 15 March 2020.^2^ Preventive measures to minimise the risk of infection were imposed, and they included regular use of surface disinfectants and the washing of hands with soap and water for at least 20 s.^3^ As an alternative to hand washing, an alcohol-based hand sanitiser with an alcohol concentration of at least 70% was recommended.^3^ With an increase in the availability and use of certain chemicals such as hand sanitisers, an increased risk of acute poisoning exposures was expected.

Previous outbreaks of respiratory viral infections such as influenza A (H1N1) in 2009, led to an increase in the use of hand sanitisers as a preventive measure against infection.^4^ This increase in use led to an increase in acute exposures as reported by poison centres in the United Kingdom (UK), New Zealand (NZ) and the United States (US).^5,6,7^ In 2020, the reports from poison centres showed a similar trend. Poison centres in the US, Croatia, Italy, Canada and France reported an increase in exposures to hand sanitiser, bleaches and other disinfectants.^8,9,10,11,12,13^ A report from the Centers for Disease Control and Prevention (CDC) and the American Association of Poison Control Centres compared National Poison Data System (NPDS) data from January 2020 to March 2020, to the same period in the preceding two years. A 20% increase in poisoning exposures to disinfectants and cleaners was seen in 2020.^8^

The effect of the COVID-19 pandemic on the telephonic calls received at a South African Poisons Information Helpline is unknown. The increased focus on sanitising along with the confinement to homes had the potential to expose people to various chemicals with a subsequent threat to their safety.^12^ Furthermore, patients often delay seeking medical care during pandemics due to the increased risk of contracting the infectious disease while at the hospital.^14^ Poison centres play a role in the prevention, diagnosis and management of poisonings and by means of toxicovigilance, identify and evaluate potential risks to human health.^15^ Furthermore, poison centres can help to prevent unnecessary hospital presentations, in the time when hospitals are overburdened by COVID-19 patients, by providing appropriate advice.^12,13^ The aim of this study was to describe all human-related calls to the Poisons Information Helpline of the Western Cape (PIHWC) during the first six months of the COVID-19 pandemic and to compare the data to similar periods in 2018 and 2019. As a secondary objective, substances that might be associated with the prevention of COVID-19 were sub-analysed. The selected substances included hand sanitisers, bleaches, essential oils and certain pharmaceuticals such as vitamins.

## Methods

A retrospective analysis was conducted on a prospectively captured observational database. The PIHWC is a joint telephone service provided by the Tygerberg Poisons Information Centre (TPIC) and Red Cross War Memorial’s Children Hospital Poisons Information Centre (RXHPIC), both situated in Cape Town, South Africa.^16^ The freely available 24/7 service provides telephonic toxicology advice to healthcare workers and members of the public. All calls are captured in real time on the electronic AfriTox TeleLog database that was established in 2015 and is registered at the Human Research Ethics Committee, University of Cape Town (R014/2014). A retrospective quality control system is in place; the entered data are firstly double-checked by a co-worker, and the suggested corrections are referred to the original data collector for consideration. The database manager makes corrections after an agreement between all involved parties is reached.

All human-related poisoning exposure calls received by the PIHWC from 01 March to 31 August during 2018, 2019 and 2020 were extracted from the database. Animal-related poisoning exposures, general poison enquiries and repeat calls were excluded. Key variables collected included the date of the call, time since exposure, province where the call originated, whether the call was received from a member of the public or a healthcare professional, patient demographics (age, gender), exposure substance, route of exposure, circumstances of exposure, symptoms reported, severity of poisoning and advice given. The poisoning severity score (PSS) was used to determine the severity of poisoning and was assigned at the time of the call. The PSS is a standardised scale used to categorise the cases of poisoning into one of five categories (none [PSS = 0], minor [PSS = 1], moderate [PSS = 2], severe [PSS = 3] and fatal [PSS = 4]).^17^

Summary statistics were used to describe all variables. Categorical data were summarised using frequency counts of percentages and distributions of variables were presented as two-way tables or bar charts. Medians or means were used as the measures of central tendency for continuous responses and standard deviations or quartiles as indications of spread. Independent proportions were compared with the ‘N-1’ chi-squared test (MedCalc Software Ltd. Comparison of proportions calculator. https://www.medcalc.org/calc/comparison_of_proportions.php [version 20.009; accessed June 25, 2021]). A 5% level of confidence was used to determine significance.

### Ethical considerations

Ethical approval was granted by the Health Research Ethical Committee, Stellenbosch University on 13 July 2021 (reference number: N20/07/039-COVID-19).

Only data from a database was used with all reference anonymised, a waiver of inform consent was part of the ethics protocol application.

## Results

**FIGURE 1:**
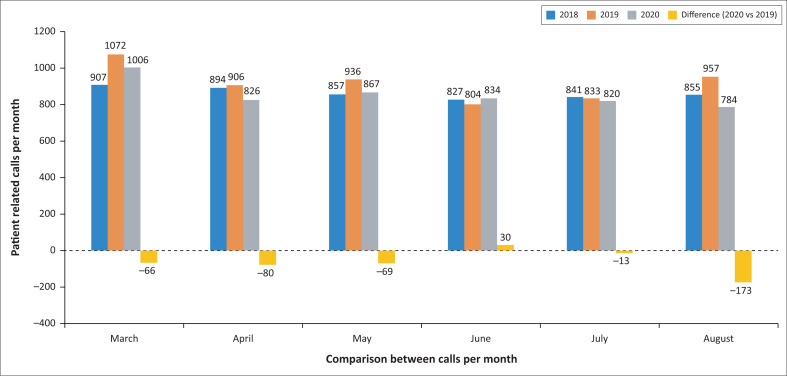
Number of calls received by the Poisons Information Helpline of the Western Cape during the first six months of the COVID-19 pandemic and similar periods during the two preceding years.

The total number of human-related calls received at the PIHWC during the study periods was 5137 in 2020, compared to 5181 in 2018 and 5508 during 2019. The number of calls per month during 2020 was mostly fewer than those observed in 2019. The main decreases in calls per month between 2020 and 2019 were experienced in August (–18.1%) and April (–8.8%) (Figure 1).

Table 1 describes the characteristics of calls to the PIHWC for the different study periods. Significantly more calls were received from the public (39.4% vs 33.1%) and for accidental exposures (65.6% vs 62.3%) during 2020 compared to 2019, while the number of intentional self-harm exposures (28.4% vs 30.2%) and therapeutic errors (3.1% vs 4.5%) decreased significantly. No statistically significant differences were observed with regard to age or gender or poisoning severity.

**TABLE 1:** Characteristics of poisoning exposure calls received by the Poisons Information Helpline of the Western Cape in the first six months of the COVID-19 pandemic and similar periods during 2018 and 2019.

Characteristics	2018		2019		2020		*p* (2020 vs. 2019)
	*n*	%	*n*	%	*n*	%	
**Caller**							
Healthcare professionals	3511	67.8	3683	66.9	3113	60.6	< 0.01
Public	1669	32.2	1825	33.1	2004	39.4	< 0.01
**Gender**							
Male	2706	52.2	2848	51.7	2677	52.1	0.68
Female	2439	47.1	2630	47.7	2425	47.2	0.58
Unknown	36	0.7	30	0.5	35	0.7	0.36
**Age (years)**							
0–5	2454	47.4	2553	46.4	2397	46.7	0.75
6–19	704	13.6	807	14.7	772	15.0	0.58
20–60	1895	36.6	1989	36.1	1804	35.1	0.29
> 60	112	2.2	150	2.7	152	3.0	0.46
Unknown	16	0.3	9	0.2	12	0.2	0.41
**Circumstances**							
Accidental	3195	61.7	3429	62.3	3371	65.6	< 0.01
Intentional self-harm	1565	30.2	1664	30.2	1461	28.4	0.05
Therapeutic error	242	4.7	248	4.5	158	3.1	< 0.01
Other/Unknown	179	3.5	167	3.0	147	2.9	0.60
**Poisoning severity**							
No symptoms(PSS = 0)	2328	44.9	2511	45.6	2350	45.8	0.60
Minor (PSS = 1)	2102	40.6	2194	39.8	2116	41.2	0.15
Moderate (PSS = 2)	536	10.4	575	10.4	478	9.3	0.05
Severe (PSS = 3)	105	2.0	122	2.2	92	1.8	0.12
Fatal (PSS = 4)	4	0.1	4	0.1	3	0.1	0.76
Unknown	105	2.0	102	1.9	98	1.9	0.83

PSS, poisoning severity score.

A total of 18 709 substances were identified: 6214 during 2018, 6535 during 2019, and 5960 during 2020. The proportions of the substance categories differed significantly between the study periods (Table 2).

**TABLE 2:** Substance categories of poisoning exposure calls received by the Poisons Information Helpline of the Western Cape in the first six months of the COVID-19 pandemic and similar periods during 2018 and 2019.

Substance category	2018		2019		2020		Difference 2020 vs. 2019		
	*n*	%	*n*	%	*n*	%	*n*	%	*p*
Antiseptic and disinfectant	251	4.0	268	4.1	430	7.2	162	3.1	< 0.001
Household chemicals†	1298	20.9	1268	19.4	1280	21.5	12	2.1	0.004
Pharmaceuticals	3110	50.0	3292	50.4	2806	47.1	−486	−3.3	< 0.001
Other‡	1473	23.7	1608	24.6	1347	22.6	−261	−2.0	0.01
Unknown	82	1.3	99	1.5	97	1.6	−2	0.1	0.65

**Total**	**6214**	**100.0**	**6535**	**100.0**	**5960**	**100.0**	**-**	**-**	**-**

† , Household chemicals: including cosmetics, household products and handyman products;

‡ , Other: Including pesticides, agricultural products, food, animal bites and stings, plants and industrial products.

The total number of exposures to pharmaceuticals decreased by 14.8% from 2020 to 2019, with four pharmaceutical subgroups being significantly different: anti-infectives (*n* = –107, –1.8%), cold and flu preparations (*n* = –87, –1.7%) and bronchodilators (*n* = –33, –0.8%) decreased, while vitamins and tonics increased (*n* = 41, 2.7%) (Appendix 1 Table 1-A1). The number of exposures to eucalyptus oil more than doubled from 2019 (*n* = 21) to 2020 (*n* = 43). No statistical difference was seen in the subgroup of anthelmintics (*n* = –1, 0.01%) that included the drug ivermectin (Appendix 1 Table 1-A1).

The exposures to antiseptics and disinfectants increased by 60.4%, mainly as a result of an increase in exposures to skin or wound antiseptics (which included hand sanitisers) (Table 2). The skin or wound antiseptic subgroup increased 67.8% from 2019 to 2020 (Appendix 1 Table 2-A1).

In the household chemicals category, a 16.5% increase in the number of bleach exposures was observed during 2020 (*n* = 191) compared to 2019 (*n* = 164).

Hand sanitiser exposures increased from 11 in 2018 and 6 in 2019 to 156 in 2020, resulting in a 26-fold increase. In 2020, 135 (86.5%) of the hand sanitiser exposures were due to alcohol-containing products. During the 2020 period, hand sanitiser exposures were mostly accidental (*n* = 118, 76.0%), while ingestion was the main route of exposure (*n* = 139, 89.0%). The exposure to hand sanitisers mainly occurred in the < 5-year age group (*n* = 66, 42.3%), followed by adults aged between 20 and 60 years (*n* = 55, 35.3%). In most exposures, no symptoms (*n* = 75, 48.1%) or minor symptoms (*n* = 72, 46.2%) were recorded. One patient was assessed as having severe symptoms (haematemesis). Gastrointestinal symptoms were most common with vomiting or nausea reported in 35 of symptomatic patients (35/81, 43.2%), and burning of the throat or stomach in 15 patients (15/81, 17.9%). Dizziness and drowsiness were also commonly reported 17/81, 20.2%). (Appendix 1 Table 3-A1). Burning and redness of the eyes were recorded in 9.9% of symptomatic patients (8/81) (Appendix 1 Table 3-A1). Of the 83 public callers in 2020, 19.3% (*n* = 16) were advised to immediately seek medical care, while 66.3% (*n* = 55) were advised to first observe the patient at home.

## Discussion

Several differences were observed in the call and substance exposure characteristics reported to the PIHWC during the first six months of the COVID-19 pandemic in 2020, when compared to similar periods in preceding years. The total number of calls received decreased, while the number of calls from the public and calls relating to accidental exposures increased. A substantial increase in exposures to hand sanitisers and essential oils was observed.

The 7% decrease in the number of calls to the PIHWC correlates with the reports from other poison centres. The Poison Control Center of Policlinico Umberto I Hospital-Sapienza University of Rome documented a 10% decrease in calls during the Italian lockdown,^12^ while a decrease was also observed in the Netherlands.^18^ This is in contrast to the Danish and French poison control centres; a 5.6% increase in the calls was documented in France.^13,18^ The lack of a common COVID-19 effect on call numbers is an indication that country-specific toxicovigilance data are needed, as the COVID-19 pandemic impacted the activity of poison centres in different ways.

Although the total number of calls to the PIHWC decreased, the number of calls from the public increased. The 6% increase in public callers in South Africa is substantially smaller than the 22% – 32% increase experienced at the poison centres in Italy, Denmark and Switzerland.^12,18^ French poison centres also documented a 14% increase.^13^ The increase is likely due to the lockdown measures implemented to minimise the contact with potentially infected persons. Under most lockdown measures, people were requested to work from home, which could thus explain the changes in type of caller. Furthermore, the fear of contracting COVID-19 in high-risk areas like hospitals and other medical facilities made poison centres a feasible option for advice, and this might also have contributed to the increase in calls from home and the public and the decrease in calls from medical professionals.

Exposures to antiseptics and disinfectants increased by 60%, showing a temporal association with increased use of these products and the COVID-19 pandemic. The percentage increase was substantially more than the 36% increase reported to the NPDS in the USA, although the actual numbers are far fewer.^8^ The increase in exposures was probably related to the recommendations from the National Institute for Communicable Diseases (NICD) of South Africa, encouraging citizens to use hand sanitisers and cleaning products as preventive measures against contracting COVID-19. The 68% increase in exposures to hand sanitisers is echoed worldwide by European and American poison centres and is likely a result of greater access to these products.^8,9,12,13,18,19^

In 46% (72/156) of the hand sanitiser exposures during the 2020 period, minor symptoms were recorded. Almost 50% of symptomatic patients had nausea or vomiting, and a further 20% were drowsy or dizzy and experienced burning of the throat or stomach. As the poison line does not routinely execute follow-ups, more severe symptoms could have developed subsequently. Most exposures occurred in the < 5-year age group (42%) and hand sanitiser ingestion in children can lead to severe hypoglycaemia and convulsions due to their high ethanol concentration. Toxic ingestion in adults can lead to respiratory and central nervous system depression as well as cardiac dysrhythmias and death.^20^ Ocular exposures were also fairly frequent,^21^ and children may be more vulnerable particularly as foot-pedal dispensers are often at the level of a child’s face.

Overall, we saw a decrease in the exposures to pharmaceuticals. Similar trends were seen in reports from the poison centres in Italy and France.^12,13^ Although various products were suggested as beneficial in the prevention and treatment of COVID-19, increase in exposure was only reported with vitamins and tonics (2.7%) and eucalyptus oil exposures that doubled from the previous year. Although it was surprising that no increase in exposures to ivermectin was seen during the study period, this likely reflects the time period for our study (March 2020 to August 2020), which was early in the COVID-19 pandemic. The recommendation is to monitor these patterns as part of the ongoing poison line toxicovigilance process. The sudden increase in eucalyptus oil exposures could have been as a result of manufacturers promoting the use of essential oils as a preventive measure against COVID-19.^22^ Similarly, vitamins and tonics might be beneficial against COVID-19 infections and were promoted as such.^23^

We acknowledge possible limitations that could have influenced the results of our study. The data are limited to voluntary reporting of exposures to poisonous substances. Although the PIHWC offers a national service, it is not known how widely the PIHWC contact details are distributed beyond healthcare facilities. The public might thus not be aware of the poison helpline and its function and might have rather contacted their local physician or emergency centre. The data are also limited to the information provided to the poison helpline by the caller, and it is not always possible to ensure complete accuracy of the described substances. The severity of poisoning was assigned at the time of the call, but cases might subsequently have become more symptomatic. Finally, experienced healthcare personnel can usually manage toxicological cases without consulting a poison line and no attempt was made to collect data from all healthcare facilities. Therefore, calls to the PIHWC might not reflect the true incidence of exposures in the country.

## Conclusion

A change in the profile of calls and exposures reported to the PIHWC was influenced by the COVID-19 pandemic. A reduction in call volume was noted during the first 6 months of the pandemic although there was an increase in the proportion of calls from the public. The increased exposures related to antiseptics and disinfectants potentially resulted from the imposed lockdown regulations and the greater availability of hygiene-related chemicals. Although the sequelae observed in these exposures were mostly mild, the public should be made aware of the dangers of exposures to these substances, particularly as small amounts can cause toxicity in children. The public should also be educated on the safe storage, proper use and potential adverse health effects of all chemicals.

## Appendix 1

**TABLE 1-A1:** Pharmaceutical exposures received by the Poisons Information Helpline of the Western Cape during the first six months of the COVID-19 pandemic and similar periods during 2018 and 2019.

Pharmaceuticals	2018		2019		2020		Difference 2020 vs 2019		
	*n*	%	*n*	%	*n*	%	*n*	%	*p*
Analgesics/anaesthetics and antipyretics	486	15.6	502	15.2	424	15.1	−78	−0.1	0.88
Antacids and ulcer remedies	29	0.9	29	0.9	22	0.8	−7	−0.1	0.67
Anthelmintics	5	0.2	9	0.3	8	0.3	−1	0.01	0.88
Anticoagulants	3	0.1	11	0.3	9	0.3	−2	−0.01	0.95
Anticonvulsants	157	5.0	159	4.8	120	4.3	−39	−0.6	0.31
Antidiarrhoeal agents	10	0.3	7	0.2	8	0.3	1	0.1	0.53
Antiemetics	18	0.6	28	0.9	22	0.8	−6	−0.1	0.76
Anti–infectives	319	10.3	392	11.9	285	10.2	−107	−1.8	0.03
Antirheumatics and gout agents	16	0.5	10	0.3	9	0.3	−1	0.02	0.89
Antispasmodics	27	0.9	25	0.8	19	0.7	−6	−0.1	0.71
Bronchodilators	57	1.8	63	1.9	30	1.1	−33	−0.8	0.01
Cardiovascular medicines	191	6.1	206	6.3	188	6.7	−18	0.4	0.49
Cold and flu remedies, antihistamines	209	6.7	273	8.3	186	6.6	−87	−1.7	0.01
Cough mixtures	41	1.3	55	1.7	35	1.2	−20	−0.4	0.18
Cytotoxics and immunosuppressants	7	0.2	4	0.1	7	0.2	3	0.1	0.23
Hormones and hypoglycaemic agents	116	3.7	108	3.3	98	3.5	−10	0.2	0.65
Laxatives	18	0.6	10	0.3	14	0.5	4	0.2	0.21
Lipid-lowering agents	16	0.5	18	0.5	19	0.7	1	0.1	0.52
Other drugs	11	0.4	15	0.5	9	0.3	−6	−0.1	0.39
Psychiatric and neurological medicines	576	18.5	579	17.6	528	18.8	−51	1.2	0.21
Skeletal muscle relaxants	22	0.7	23	0.7	29	1.0	6	0.3	0.16
Sleeping pills	148	4.8	127	3.9	100	3.6	−27	−0.3	0.54
Slimming preparations	9	0.3	10	0.3	14	0.5	4	0.2	0.21
Substance abuse	61	2.0	63	1.9	46	1.6	−17	−0.3	0.43
Topicals (creams, drops, oral preps)	264	8.5	280	8.5	262	9.3	−18	0.8	0.26
Traditional medicines	9	0.3	5	0.2	3	0.1	−2	0.0	0.67
Unknown drugs	33	1.1	41	1.2	31	1.1	−10	−0.1	0.59
Vitamins, minerals, tonics	252	8.1	240	7.3	281	10.0	41	2.7	< 0.01

**Total**	**3110**	**100.0**	**3292**	**100.0**	**2806**	**100.0**	**-**	**-**	**-**

**TABLE 2-A1:** Antiseptic and Disinfectant exposures received by the Poisons Information Helpline of the Western Cape in the first six months of the COVID-19 pandemic and similar periods during 2018 and 2019.

Antiseptics/disinfectants	2018		2019		2020		Difference 2020 vs 2019		
	*n*	%	*n*	%	*n*	%	*n*	%	*p*
Environmental disinfectants	73	29.1	63	23.5	89	20.7	26	−2.8	0.38
Skin or wound antiseptics	177	70.5	202	75.4	339	78.8	137	3.4	0.3
Unknown antiseptics	1	0.4	3	1.1	2	0.5	−1	−0.6	0.37

**Total**	**251**	**100**	**268**	**100**	**430**	**100**			

**TABLE 3-A1:** Common symptoms following hand sanitiser exposure recorded by the Poisons Information Helpline of the Western Cape during the first six months of the COVID-19 pandemic.

Symptom	Number	% of symptomatic patients
Nausea or vomiting	35	43.2
Drowsiness or dizziness	17	21.0
Burning throat/stomach	15	18.5
Eyes - red/burning	8	9.9
Shortness of breath	3	3.7
Slurred speech	2	2.5
Seizures	2	2.5
Headache	2	2.5

*N* = 81.
